# The polyketide synthase PKS15 has a crucial role in cell wall formation in *Beauveria bassiana*

**DOI:** 10.1038/s41598-020-69417-w

**Published:** 2020-07-28

**Authors:** Somsiri Udompaisarn, Wachiraporn Toopaang, Udom Sae-Ueng, Chettida Srisuksam, Nuchnudda Wichienchote, Rudsamee Wasuwan, Nur Amalina Shamsun Nahar, Morakot Tanticharoen, Alongkorn Amnuaykanjanasin

**Affiliations:** 1 National Center for Genetic Engineering and Biotechnology (BIOTEC), National Science and Technology Development Agency (NSTDA), 113 Thailand Science Park, Paholyothin Rd., Tambon Khlong Nueng, Amphoe Khlong Luang, 12120 Pathum Thani Thailand; 2 School of Bioresources and Technology, King Mongkut’s University of Technology Thonburi, Bangkok, 10140 Thailand; 3 Temasek Polytechnic, 21 Tampines Avenue 1, Singapore, 529757 Singapore

**Keywords:** Cell growth, Fungal genes, DNA recombination

## Abstract

Entomopathogenic fungi utilize specific secondary metabolites to defend against insect immunity, thereby enabling colonization of their specific hosts. We are particularly interested in the polyketide synthesis gene *pks15*, which is involved in metabolite production, and its role in fungal virulence. Targeted disruption of *pks15* followed by genetic complementation with a functional copy of the gene would allow for functional characterization of this secondary metabolite biosynthesis gene. Using a *Beauveria bassiana* ∆*pks15* mutant previously disrupted by a bialophos-resistance (*bar*) cassette, we report here an in-*cis* complementation at *bar* cassette using CRISPR/Cas9 gene editing. A *bar*-specific short guide RNA was used to target and cause a double-strand break in *bar*, and a donor DNA carrying a wild-type copy of *pks15* was co-transformed with the guide RNA. Isolate G6 of *∆pks15* complemented with *pks15* was obtained and verified by PCR, Southern analyses and DNA sequencing. Compared to ∆*pks15* which showed a marked reduction in sporulation and insect virulence, the complementation in G6 restored with insect virulence, sporulation and conidial germination to wild-type levels. Atomic force and scanning electron microscopy revealed that G6 and wild-type conidial wall surfaces possessed the characteristic rodlet bundles and rough surface while ∆*pks15* walls lacked the bundles and were relatively smoother. Conidia of ∆*pks15* were larger and more elongated than that of G6 and the wild type, indicating changes in their cell wall organization. Our data indicate that PKS15 and its metabolite are likely not only important for fungal virulence and asexual reproduction, but also cell wall formation.

## Introduction

*Beauveria bassiana*, an entomopathogenic fungus, has a broad host spectrum and is considered to have high potential for insect biocontrol in agriculture. While *B. bassiana* can cause mycosis in several insect species^1,2^, insect killing is fairly slow due to several limiting factors, particularly in the field. The fungus is also vulnerable to environmental stress factors such as UV radiation, high temperature and drought^3^. A better understanding of the biological and physiological characteristics of this entomopathogen should allow us to improve its virulence and stress tolerance.

Secondary metabolites are abundant in entomopathogenic fungi and include polyketides, nonribosomal peptides, terpenes and alkaloids that play important roles in various aspects of the fungal life cycle. *B*. *bassiana* BCC2660, a widely used biocontrol fungus in Thailand, has 12 polyketide synthase (PKS) genes in its genome^4^. Two PKS genes, *pks15* and *pks14*, have crucial roles in virulence against insects, as previously demonstrated by targeted gene deletion^4,5^. The *pks15* mutant exhibits loss in phagocytic survival ability, a phenotype likely associated with changes in the cell wall, the outermost layer of fungal conidia. Unfortunately, little is known regarding the relationship between polyketides and the fungal wall. In a few reports, melanin, the metabolite of a non-reducing PKS and other enzymes in melanin biosynthetic pathway, has been found in the cell walls of *Aspergillus fumigatus*^6^, *Colletotrichum lagenarium*^7^, *Neurospora crassa*^8^ and *Pestalotiopsis microspora*^9^. The *P. microsopora* PKS1 is also important for wall integrity and conidial germination in this endophytic fungus^9^. In addition, the green pigment citreoisocoumarin, synthesized by a non-reducing PKS, is present in the cell wall of mature *A. nidulans* conidia^10,11^. To the best of our knowledge, however, no report thus far has identified a role for a reducing PKS in modulating the fungal wall.

Previously, using a gene knock-out approach, we found that insect virulence of the *B. bassiana pks15* deletion mutant was considerably reduced^4^. Furthermore, our preliminary observation suggested that the spore wall surface of Δ*pks15* was noticeably different from that of the wild type. In this study, we have set out to investigate whether PKS15 has impact on the fungal wall by performing genetic complementation of Δ*pks15* by CRISPR/Cas9 to verify the role of PKS15 in cell wall formation.

So far, there have been a few reports on *B. bassiana* genome editing of using the CRISPR/Cas9 approach. This clustered regularly interspaced short palindromic repeat sequence (CRISPR)/Cas9 system has been developed to perform genome editing in various microorganisms^12^. This system consists of two components: the Cas9 endonuclease and a single chimeric guide RNA (sgRNA) containing 20-nucleotides matching the target DNA region followed by protospacer adjacent motif (PAM). Base pairing of the sgRNA and the target DNA results in recruitment of Cas9 to generate a specific double-strand break (DSB)^13^. DSBs can be repaired by either error-free homologous recombination called homology-directed repair (HDR) in the presence of a donor DNA or error-prone repair via non-homologous end-joining (NHEJ). The latter usually leads to a small insertion or deletion in the target sequence, resulting in gene mutation.

Here, we employed CRISPR/Cas9 with HDR to obtain a complemented isolate of *B. bassiana* Δ*pks15* with a functional *pks15*. The complemented isolate was verified by PCR and Southern analyses and examined various phenotypes, namely sporulation, germination, We report the impact of PKS15 in the cell wall formation in this study.

## Results

### *Directed mutagenesis of the* bar *cassette in the* Δpks15 *mutant efficiently driven by NHEJ repair*

To study the function of PKS15 in *B. bassiana* BCC 2660, we were interested in generating a PKS15-complemented *∆pks15* mutant by CRISPR/Cas9. However, the CRISPR/Cas9 vector used in this study was previously developed for *Aspergillus aculeatus*^13^, so we first tested this vector for its ability to mediate targeted genome editing in the *B. bassiana* BCC 2660 *∆pks15* mutant. The sgRNA target site was designed based on the nucleotide sequence of the *bar* cassette previously inserted within *pks15* to disrupt gene expression^4^. A list of candidate *bar* protospacer sequences was generated using a web-based sgRNA analysis tool (https://bioinfo.imtech.res.in/manojk/gecrispr/index.php). The sequence with the lowest likelihood of off-target binding was selected.

Construction of the *bar*-targeting vector, pCas9sgBar, was performed by introducing the protospacer into the CRISPR/Cas9 backbone vector via USER cloning^13^. DNA sequencing was used to verify sequence integrity and the correct sequential order of pCas9sgBar elements. The elements of pCas9sgBar are shown in Supplemental Fig. S1. The vector pCas9sgBar was then transformed into the *pks15* mutant using PEG-mediated protoplast transformation. Transformants were first screened for hygromycin resistance conferred by pCas9sgBar. Additionally, as the *bar* cassette enables growth on medium supplemented with glufosinate^4^, hygromycin-resistant transformants were subsequently screened for the inability to grow in the presence of glufosinate, which would indicate successful mutation of the *bar* cassette. Among 50 hygromycin-resistant transformants, two clones, namely A26 and C1, had lost glufosinate resistance (data not shown). Single spore isolation was performed to purify those mutants, and those isolates were again verified for hygromycin resistance and glufosinate sensitivity.

To determine the sequence of *bar* in A26 and C1, their *bar* fragments were amplified with a pair of *bar*-specific primers, cloned into the TA cloning vector pCR 2.1, and submitted for DNA sequencing. Sequencing results showed that clones A26 and C1 both had a one-base insertion in the *bar* cassette (Table 1). These genome alterations resulted in a frameshift mutation in *bar*, changing their amino acid sequences. The CRISPR/Cas9 system, therefore, can mediate targeted gene editing in *B. bassiana* BCC 2660.

**Table 1:** Mutations in the *bar* locus of three mutants. The mutants A26 and C1 were directly mutagenized by CRISPR/Cas9.

	DNA sequence	Insertion\deletion	Amino acid sequence
	*bar*-targeting sgRNA		
*∆pks15* (with a functional *bar* cassette)	5′..ACCCACCTGCTGAAGTCCCTGGAGGCACAG..3′		..THLLKSLEAQ.
A26 *bar* mutant	5′..ACCCACCTGCTGAAGTCCC***T*** TGGAGGCACAG..3′	**+ 1**	..THLLKSL**GGT**.
C1 *bar* mutant	5′..ACCCACCTGCTGAAGTCCC***C*** TGGAGGCACAG..3′	**+ 1**	..THLLKS**PGGT.**

The one-base insertions in A26 and C1, highlighted in italics, caused frame shifts and changes in the amino acid sequence. The PAM site is underlined.

### *In-cis genetic complementation of* Δpks15 *using CRISPR/Cas9*

**Figure 1:**
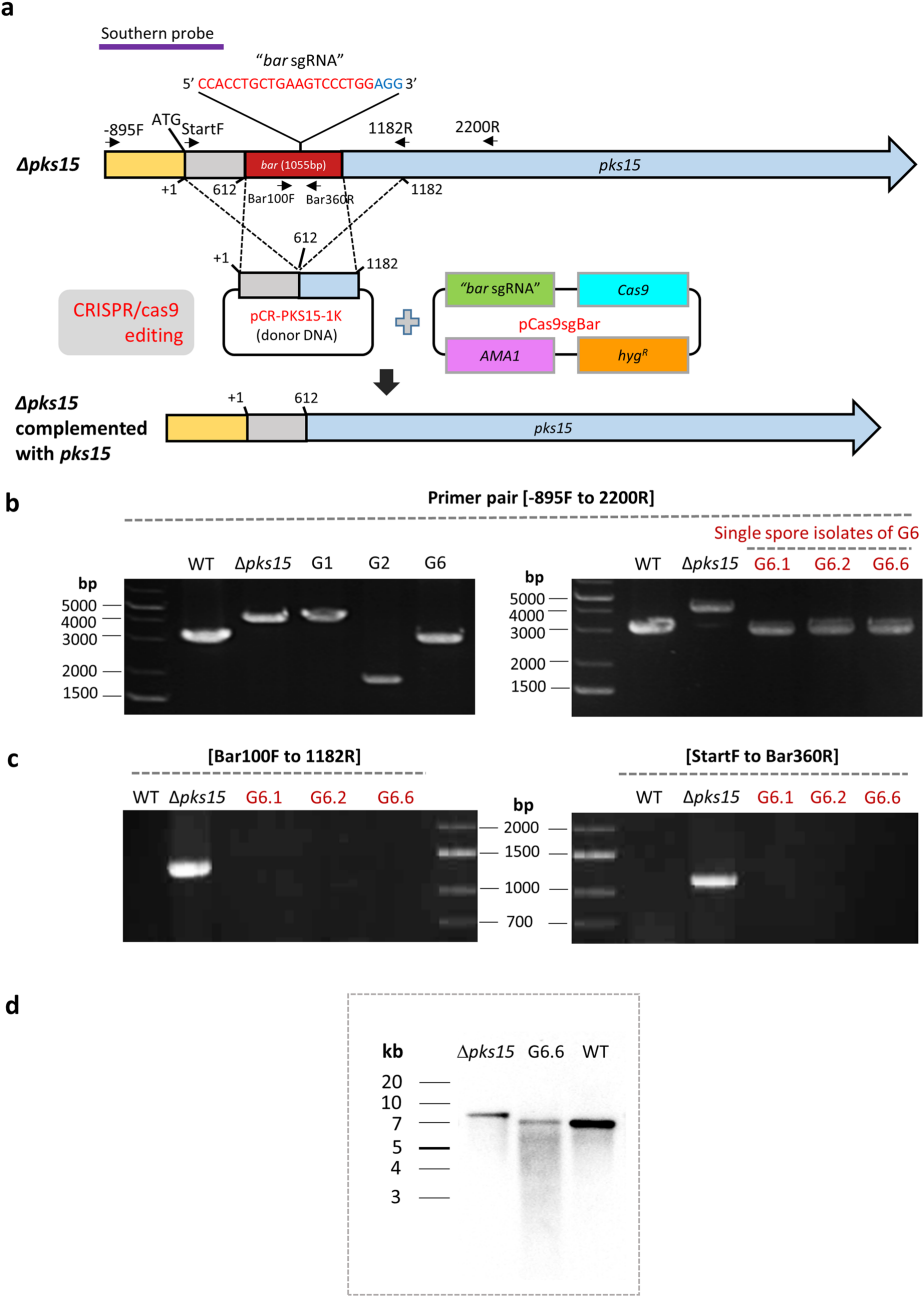
(**a**) A schematic diagram of CRISPR/Cas9-mediated genome editing in the ∆*pks15* mutant by targeting the selection marker gene *bar* that was used to disrupt *pks15*. An in-*cis* complementation of the ∆*pks15* mutant was performed by homologous recombination with a wild-type copy of *pks15* using the donor DNA pCR-PKS15-1K. Molecular analysis of the *pks15* locus of transformants G1, G2 and G6 (from genetic complementation of ∆*pks15*) was compared to those for the wild type (WT) and *Δpks15* using PCR (**b, c**) and Southern (**d**) analyses. Primers used for PCR and their locations are shown in (**a**). (**b**) PCR amplification with primers PKS15-minus-895F and PKS15-2200R primer. (**c**) PCR amplifications with primers Bar100F and PKS15-1182R, and PKS15-StartF and Bar360R. (**d**) Southern blotting results for *Δpks15*, the complemented isolate G6.6 and the wild type. Genomic DNA was digested with *Eco*RI and hybridized with a *pks15*-specific probe, shown in (**a**).

In addition to directed mutagenesis, the CRISPR/Cas9 system has the potential to mediate targeted genetic complementation via HDR. In Δ*pks15*, the *bar* cassette has been integrated in *pks15*, thereby disrupting gene function^4^. To further verify the role of *pks15* in the fungus, a circular DNA donor pCR-PKS15-1k, which has a 1.0 kb *pks15* sequence serving as arms for homologous recombination, was co-transformed with pCas9sgBar into *∆pks15* for homologous replacement of *bar* (Fig. 1a).

**Figure 2:**
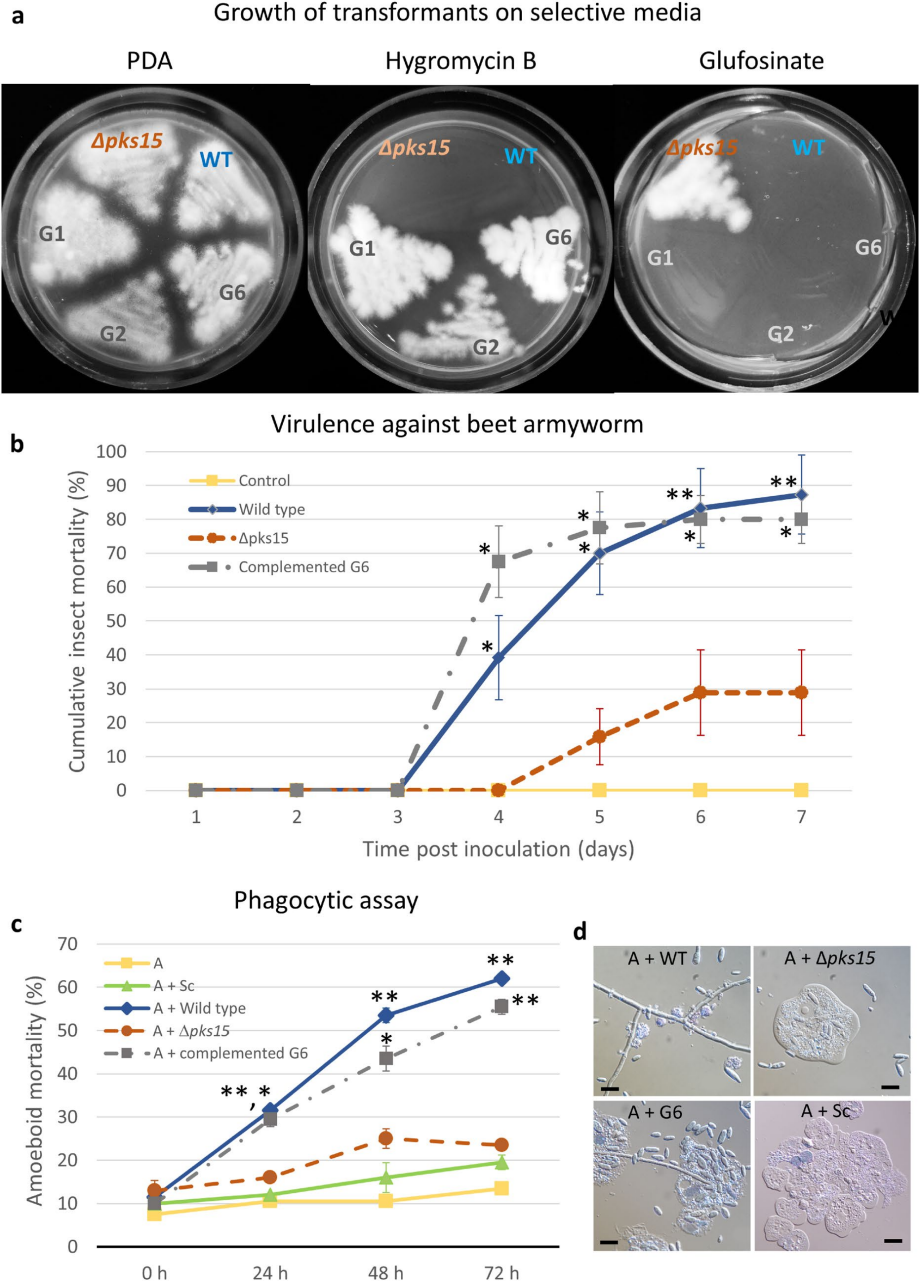
(**a**) Growth of the *B. bassiana* wild type (WT), *Δpks15* and transformants G1, G2 and G6 (from genetic complementation of ∆*pks15*) on PDA with/without hygromycin B or on a minimal medium containing glufosinate. The three isolates G1, G2 and G6 were resistant to hygromycin B but became sensitive to glufosinate. (**b**) Virulence against beet armyworm larvae determined by cumulative insect mortalities (%) caused by the *B. bassiana* wild type*,* Δ*pks15* and complemented isolate G6 using a low-dose inoculum (1 × 10^5^ conidia ml^−1^). Saline was used as the control. (**c**) Phagocytic assay using the soil amoeba *A. castellanii*. Mortality rates (%) of the amoebae (*A*) after incubation with blastospores of the *B. bassiana* wild type, ∆*pks15* and the complemented isolate G6 and *S. cerevisiae* cells (control). Data shown are mean ± S.E.M. Asterisks indicate statistical significance between the wild type or the complemented isolate G6 and Δ*pks15* (Student’s *t* test: *p < 0.05; **< 0.01). (**d**) Light micrographs of amoebae (*A*) after incubation with blastospores of the *B. bassiana* wild type, ∆*pks15* and G6 and *S. cerevisiae* cells for 24 h. The wild type and G6 grew to generate hyphae at this early time point. Bars, 10 µm.

In the genetic complementation of *∆pks15*, three transformants (G1, G2 and G6) grew on medium supplemented with hygromycin, but failed to grow on the glufosinate-supplemented one. This indicated the hygromycin resistance and glufosinate sensitivity of the aforementioned transformants (Fig. 2a). We performed molecular analysis of the *pks15* locus in transformants G1, G2 and G6 by PCR amplification, in comparison to that of the wild type and Δ*pks15*. The primers’ locations are mapped in Fig. 1a. G6 gave an amplification product of 3.2 kb similar to the wild-type amplicon, whereas G1 and G2 did not (Fig. 1b, left panel). The transformant G6, identified as a complemented isolate of Δ*pks15*, was selected for further experiments and subjected to single spore isolation. It should be noted that, although we did not obtain a high number of transformants in our complementation experiment, one out of three (33%) had correctly repaired the *pks15* locus. This recombination frequency is markedly higher than those of homologous recombination performed by conventional, non-CRISPR methods in various *B*. *bassiana* strains. For instance, 7–13% recombination was observed for strain Bb0062^14^ and 7–25% for our BCC 2660 strain^4,5^. After single spore isolation, all ten isolates were re-checked for hygromycin and glufosinate resistance as described above. Three single spore isolates, G6.1, G6.3 and G6.6, were randomly selected for PCR analysis. Those three isolates gave products of similar size as the wild type (Fig. 1a, right panel). Furthermore, we checked for the presence of *bar* cassette using the *bar*-specific primers. The *pks15* mutant was the only one able to produce *bar*-derived PCR products, whereas isolates G6.1, 6.3, 6.6 and the wild type did not (Fig. 1c). Southern hybridization also showed that isolate G6.6 had a hybridized band similar to that of the wild type and noticeably shorter than that of Δ*pks15* (Fig. 1d). The PCR and Southern hybridization data confirmed that the *bar* cassette had been removed in the complemented isolate G6. Lastly, for the molecular analysis of the *pks15* locus, DNA sequencing demonstrated the sequence integrity of *pks15* in G6.6, being identical to that of the wild type (Supplemental Fig. S2). Together, these results indicated that replacement of the *bar* cassette with a wild-type copy of *pks15* via CRISPR/Cas9-induced HDR was successful in this entomopathogenic fungus.

### Restoration of wild-type levels of sporulation and germination in the complemented isolate

The sporulation assay has shown that Δ*pks15* produced significantly fewer conidia and blastospores than the wild type, as we previously reported^4^. In this study, the complemented isolate G6 restored sporulation, with an increase in the percentage of conidia (37–88%) and blastospores (30–98%) compared to Δ*pks15*, relative to wild-type levels (Table 2). Conidial germination was also restored from 34% in Δ*pks15* to 77% in the complemented isolate G6 (Table 2).

**Table 2:** Comparative sporulation and germination of the *B. bassiana* wild type, *Δpks15* and the complemented isolate G6.

Strains	Relative sporulation (%)		Conidial germination (%)
	Conidia	Blastospores	
Wild type	100*	100*	74* ± 6.0
Δ*pks15*	30.5 ± 1.0	37.5 ± 1.3	34 ± 3.1
Complemented isolate G6	98.7* ± 4.8	88.9* ± 2.2	77* ± 4.0

Relative sporulation (%) was determined by the number from spores in each strain relative to that of the wild type. Conidia and blastospore yields were determined on 5-day-old PDA and in 2-day-old SDY broth, respectively. Germination analysis was performed by incubation of conidia in 5% (v/v) PDB for 20 h.

Data shown are mean ± S.E.M. Asterisks indicate statistical significance relative to that of Δ*pks15* (Student’s *t*-test, *p* < 0.05).

### Restoration of insect virulence and phagocytic survival ability of the complemented isolate

Virulence against the beet armyworm was determined by inoculating low doses (300 conidia) of the wild type, Δ*pks15* or the complemented isolate G6 into larvae. Mortality data showed that the complemented isolate G6 resulted in similar insect mortalities to that of wild type over the observation period (Fig. 2b). On day 4 post-injection, mycosis and insect death were clearly observed in the worms inoculated by the wild type and the complemented isolate G6, resulting in 30% and 56% mortality, respectively (Fig. 2b). On the other hand, insect larvae injected with Δ*pks15* exhibited only 10% insect mortality at the same time point. At the end of experiment (day 7 post-injection), the mortality rate of worms inoculated with the wild type and complemented isolate G6 was at 95%, whereas Δ*pks15*-associated mortality was at 60%. The mean lethal time (LT_50_) of insect larvae injected with the wild type, Δ*pks15* and complemented isolate G6 was 4.09, 4.71 and 3.95 days, respectively. Thus, the complemented isolate G6 was capable of killing larvae at a rate similar to that of the wild type. These data indicated that the impaired insect virulence of Δ*pks15* could be restored by complementation with a wild-type copy of *pks15*, thus further cementing a role for *pks15* in insect virulence.

We previously investigated whether PKS15 mediated the first line of insect host defense by assessing the ability of the wild type and Δ*pks15* in escaping phagocytosis using the soil-dwelling amoeba *Acanthamoeba castellanii* as a model of study. We found that phagocytic survival was impaired in Δ*pks15* compared to the wild type^4^. Here, we studied G6 in a similar assay and conducted microscopy to monitor amoeboid mortality. Similar to the previous result, several blastospores of Δ*pks15* were engulfed and lysed by the amoeba, whereas most wild-type and G6 blastospores escaped from phagocytosis (Fig. 2d). These blastospores then propagated extensively and switched to a vegetative phase, eventually leading to amoeboid death. Our quantitative data showed that co-culture of wild-type and G6 blastospores with phagocytic amoeba resulted in 40–50% amoeba mortality, while only ~ 20% mortality was seen for Δ*pks15* at 48 and 72 h after mixing (Fig. 2c). This indicated that the ability of the complemented isolate G6 to counteract with phagocytosis was restored to wild-type levels.

### PKS15 is involved in cell wall formation

**Figure 3:**
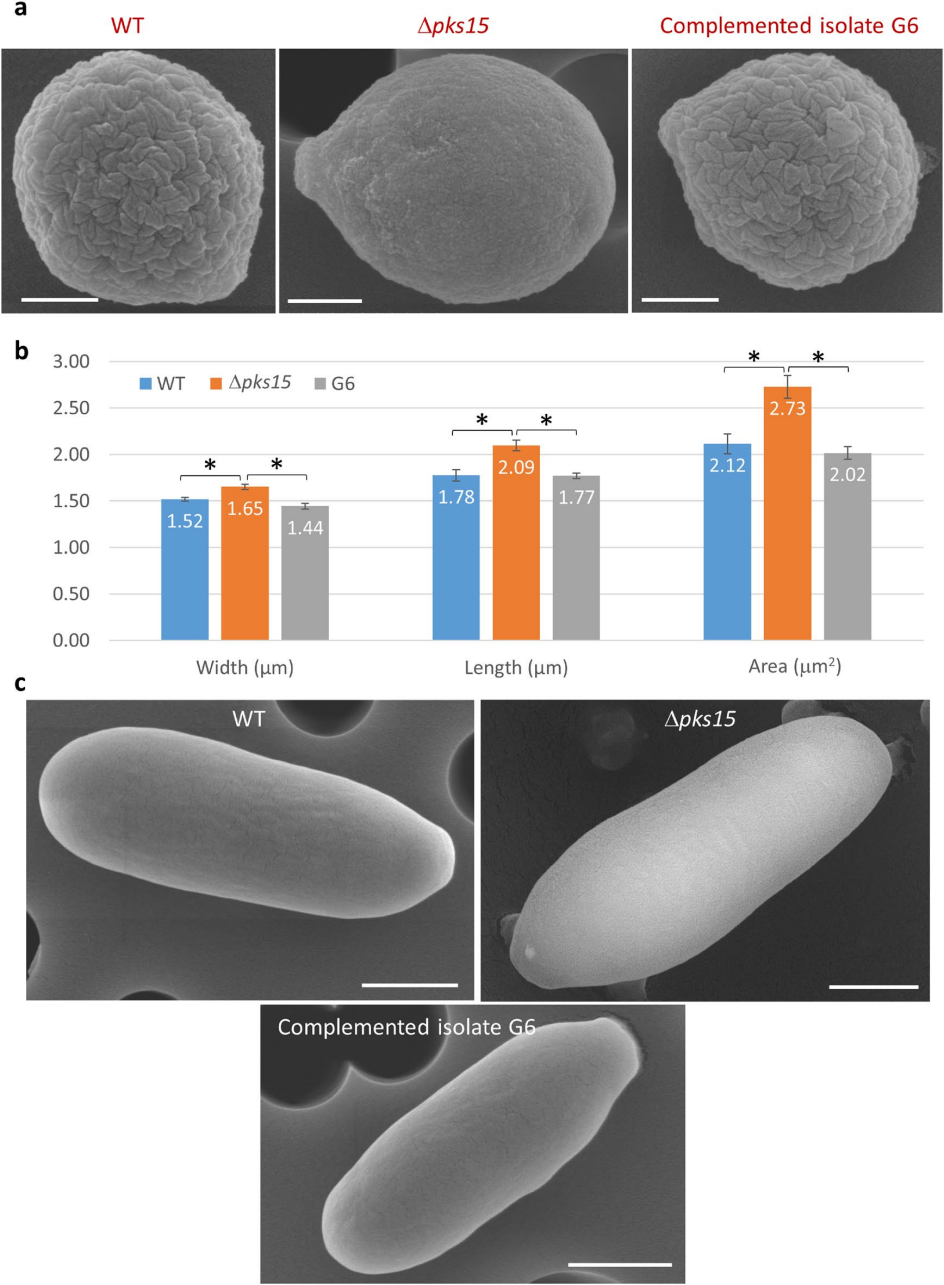
(**a**) Scanning electron micrographs (SEMs) of conidia from the wild type (WT), Δ*pks15* and the complemented isolate G6. It is noted that wild-type and G6 conidia have characteristic rodlet bundles on the wall surface but the *pks15* mutant lacks these bundles. Also, this Δ*pks15* conidium, as a representative of most of the mutant conidia, is larger and more elongated than that of the wild type and G6. Bars, 500 nm. (**b**) Measurement of width, length and area of conidia from the three strains, as analyzed from the electron micrographs taken. Data shown are mean ± S.E.M. Asterisks indicate statistical significance between the wild type or the complemented isolate G6 and Δ*pks15* (Student’s *t* test: *p < 0.05). (**c**) SEMs of blastospores from WT, Δ*pks15* and G6. Bars, 1 µm.

Since the cell wall is the outermost part of the cell which actively interacts with the surrounding cells, we thus explored the cell wall surface characteristics in the wild type, *pks15* mutant and G6. We performed a series of comparative analyses on cell wall characterization used scanning electron microscopy (SEM) to determine the cellular characteristics of conidia used in the insect bioassay and blastospores. The appearance of rodlet bundles on the cell wall surface was the most striking difference seen with the conidia of the wild type and G6 compared to Δ*pks15* (Fig. 3). Wild-type and G6 conidia had rough wall surfaces and smaller sizes, while mutant conidia had smoother surfaces and larger sizes. Size measurements derived from the scanning electron micrographs indicated that Δ*pks15* conidia were significantly larger in width, length and size (area) than the wild type and G6 (Fig. 3a,b). The average size of Δ*pks15* conidia was 1.65 × 2.09 μm (width and length) compared to 1.52 × 1.78 and 1.44 × 1.77 μm for the wild type and G6, respectively (Fig. 3b). The Δ*pks15* conidia appeared as an ellipse-like shape, compared to circular forms seen for the other two strains.

**Figure 4:**
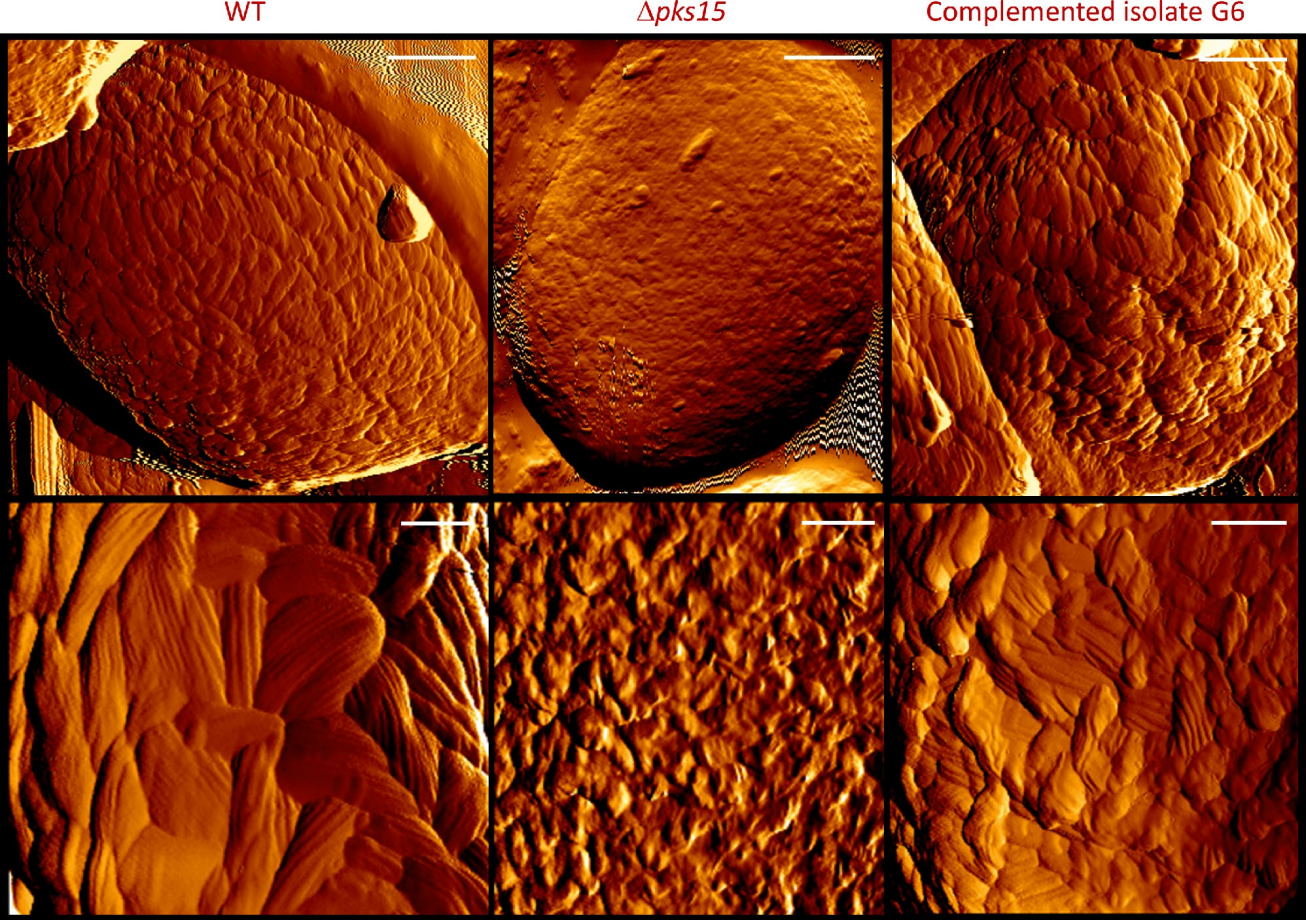
Atomic force micrographs of conidial surfaces from the wild type (WT), Δ*pks15* and the complemented isolate G6. Amplitude images are shown. Rodlet bundles were found on the surface of wild-type and G6 conidia, but not detected for Δ*pks15*. Bars, 500 and 100 nm for upper and lower panels, respectively.

Atomic force microscopy (AFM) was also performed to visualize conidial wall surface. It revealed a dramatic difference between the wild type and the complemented isolate G6 and that of Δ*pks15*, with wild-type and G6 walls clearly possessing characteristic rodlet bundles, whereas Δ*pks15* walls lacked such a feature (Fig. 4). However, the arrangement of bundles in the wild type appeared to be more uniform throughout the conidial surface than that of G6, which revealed a few bulges on the surface. The typical bundles disappeared or drastically reduced in size in the Δ*pks15* conidial surface, with rodlets barely visible. Quantitative analysis of the rodlets showed that the wild type and G6 had similar average numbers of rodlets in each bundle (6.7 ± 1.4 and 6.1 ± 1.3, respectively).

**Figure 5:**
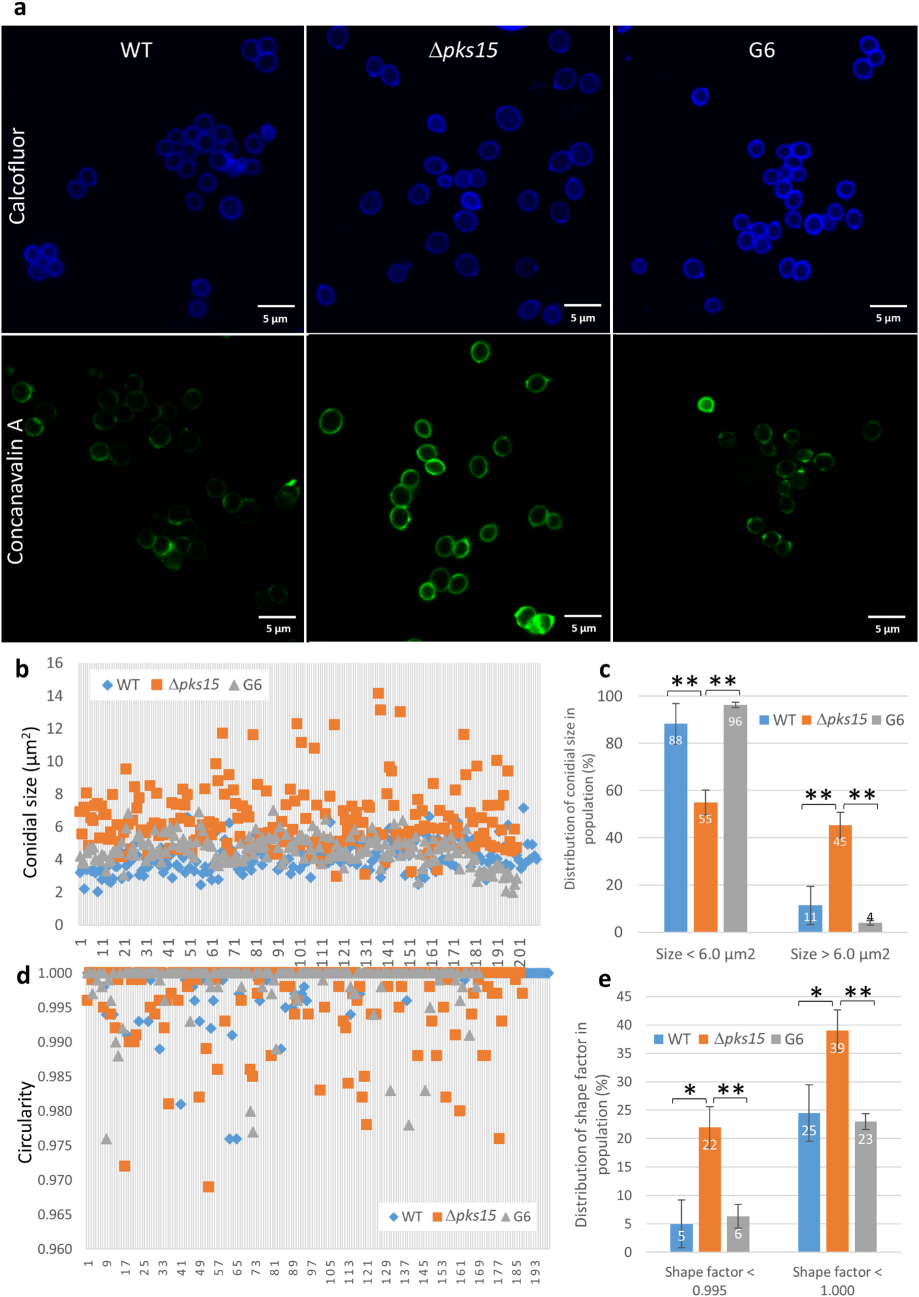
Size and shape of fluorescently-stained conidia in the wild type (WT), Δ*pks15* and the complemented isolate G6. (**a**) Calcofluor- and FITC-tagged concanavalin A staining (upper and lower panels). Bars, 5 μm. (**b**) Distribution of sizes in the conidial populations of the wild type, Δ*pks15* and G6 from a single representative experiment. (**c**) Frequency of conidial sizes for each of the three strains. Size data were from three independent experiments. (**d**) Distribution of shapes in the conidial populations of wild type, Δ*pks15* and G6 from a single representative experiment. Shape factor (circularity) was determined using the NIS-Elements D software. A shape factor of 1.0 indicates a circle, whereas a shape factor less than 1.0 indicates an ellipse. (**e**) Frequency of conidial shapes for each of the three strains. Data shown are mean ± s.e.m. Asterisks indicate statistical significance between the wild type or the complemented isolate G6 and Δ*pks15* (Student’s *t*-test: *p < 0.05; **< 0.01).

**Figure 6:**
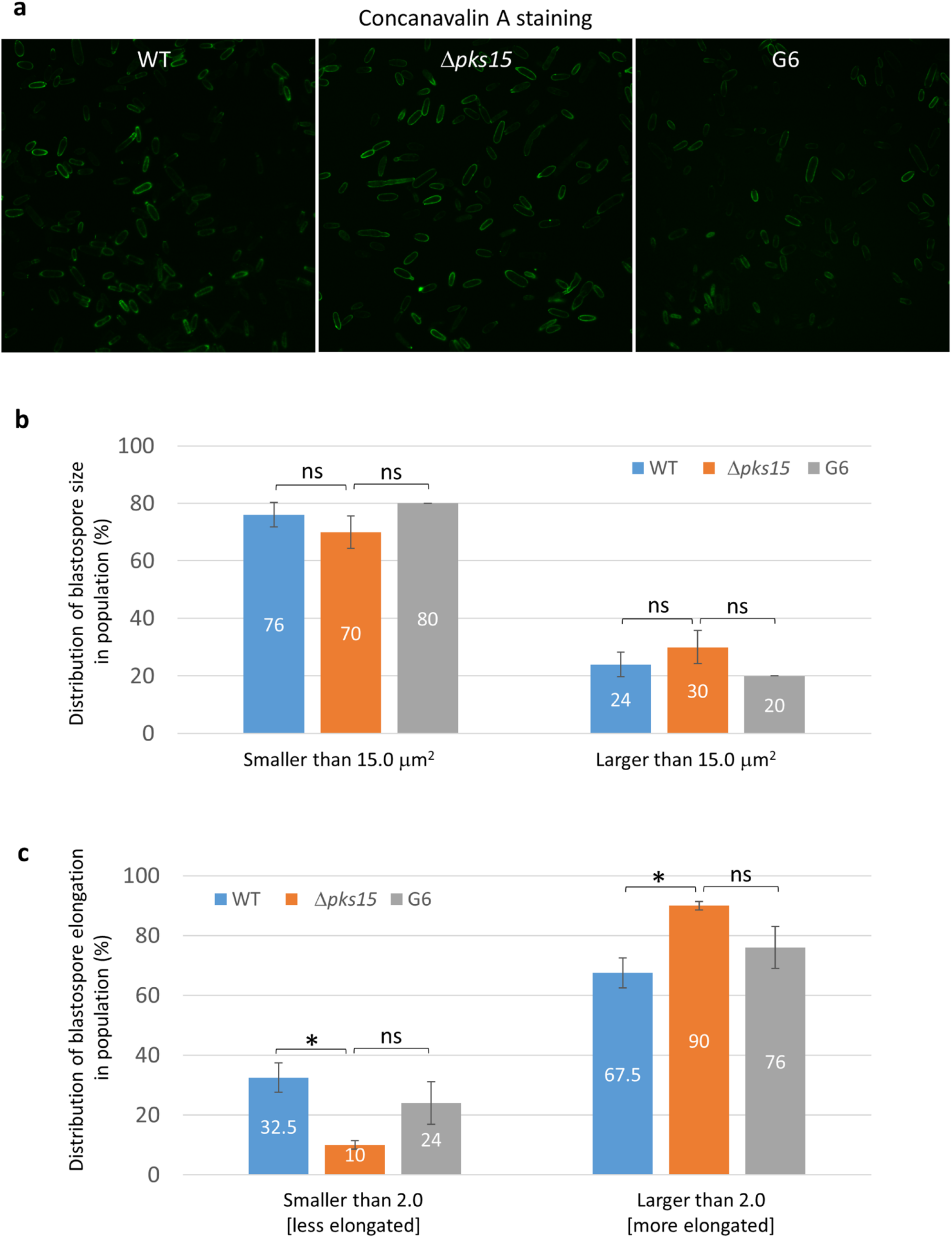
Size and shape of fluorescently-stained blastospores in the wild type (WT), Δ*pks15* and the complemented isolate G6. (**a**) FITC-tagged concanavalin A staining. Bars, 5 μm. (**b**) Frequency of blastospore sizes for each of the three strains. (**c**) Frequency of blastospore elongation for each of the three strains. Data shown are mean ± S.E.M. Asterisks indicate statistical significance between the wild type or the complemented isolate G6 and Δ*pks15* (Student’s *t*-test: *p < 0.05; *ns* not significant).

Lastly, we examined differences in cell wall carbohydrate characteristics by staining conidia and blastospores with concanavalin A, which binds to α-glucan and α-mannan, and calcofluor white, which binds to chitin. Concanavalin A-stained Δ*pks15* conidia, and blastospores to a lesser extent, exhibited brighter staining patterns compared to wild-type and G6 blastospores under identical staining and microscopic protocols (Figs. 5a, 6a). In contrast, the calcofluor-stained wild type, Δ*pks15* and G6 conidia (Fig. 5a) as well as blastospores (data not shown) displayed similar fluorescence intensity.

We also determined the size and cell characteristics of stained conidia and blastospores. From 2D images, overall conidial size (areas in 2D) of Δ*pks15* was larger than that of the wild type and complemented isolate G6 (Fig. 5b,c), similar to SEM analysis results. In the wild type and complemented isolate G6, more than 88% of conidia were smaller than 6.0 µm^2^. In contrast, the ratio of Δ*pks15* conidia with areas less than to greater than 6.0 µm^2^ was 55:45. A number of Δ*pks15* conidia were bigger than 10 µm^2^, which is unusual and strikingly larger than typical *B. bassiana* conidia. We then applied the shape factor (circularity) feature in the NIS-Elements D software version 5.10 (Nikon, USA) with the formula [Shape Factor = 4 π (Area)/(Perimeter)^2^] to analyze conidia shapes. Nearly 40% of the Δ*pks15* conidial population had an elliptical shape rather than the circular form mostly seen with the wild type and G6 (Fig. 5d,e).

Comparisons of blastospore shapes and sizes did not reveal as large a difference among the three strains. The Δ*pks15* blastospores were noticeably smaller than the wild type and G6, however. When blastospores were classified as either smaller or larger than 15.0 μm^2^, Δ*pks15* blastospores exhibited a 70:30 ratio compared to 76:24 and 80:20 for the wild type and G6, respectively (Fig. 6b). Nonetheless, the differences were not statistically different (*p* > 0.05). With respect to the shape of blastospores, we assessed their elongation values using the formula [Elongation = MaxFeret/MinFeret]. The Δ*pks15* blastspores were more elongated than that of the wild type and G6, with nearly all (90%) Δpks15 blastospores being more elongated (elongation value of > 2.0) compared to 67–76% for wild type and G6 blastsopores (Fig. 6b). In contrast, only 10% of Δpks15 blastspores were less elongated (elongation value of < 2.0), compared to 33% and 24% for the wild type and G6 (Fig. 6c), respectively.

## Discussion

In our previous report, the gene *pks15* was observed to be expressed in almost all culture conditions tested for *B. bassiana*^15^, including potato dextrose broth (PDB) (where conidia are produced) and Sabouraud dextrose broth supplemented with 1% yeast extract (SDY) (where in vitro blastospores are formed). We thus hypothesized that PKS15 could be important for fungal growth and development. Indeed, PKS15 is necessary for the formation of conidia and blastospores in this fungus^4^. SEM and AFM examination of *B. bassiana* conidia in the current study revealed a remarkable difference in the cell walls, with conidial wall surfaces in the wild type and complemented isolate G6 appearing rough and possessing fascicle bundles of the wall rodlet layer. In contrast, the *pks15* mutant’s conidial wall surface was noticeably smoother and completely lacked the bundles. Since the rodlet bundle is mainly composed of hydrophobin in *B. Bassiana*^16^, PKS15 could be linked to the presence or assembly of hydrophobin in the cell wall. Nevertheless, the *pks15* mutant is not entirely similar to the hydrophobin mutants Δ*hyd1* and Δ*hyd1*Δ*hyd2*. These two mutants exhibit increased conidial germination compared to the wild type^16^. In contrast, a noticeably lower percentage of Δ*pks15* conidia germinate compared to the wild type^4^. Thus, there could be other components in the cell wall or within the cell that are mediated by PKS15 functions.

Wall carbohydrate staining indicated that there was a marked difference in the fluorescence intensity of concanavalin A staining between the wild type and G6 on the one hand and Δ*pks15* on the other. This suggests an alteration of mannan and glucan (substrates of concanavalin A binding) organization in the wall of the *pks15* mutant. Alternatively, it is possible that target substrates could be more accessible to the corresponding dyes in Δ*pks15* compared to the wild type and G6 due to the lack of rodlet bundles in Δ*pks15*. Furthermore, the size and shape of the stained conidia were apparently affected by *pks15* disruption. Here, we revealed that Δ*pks15* conidia were significantly larger and more elongated than the wild type and G6. The Δ*pks15* blastospores were also more elongated. Together, our wall analysis data showed that *pks15* disruption results in changes in the spore wall architecture, including rodlet bundle formation and spore size and shape, leading us to surmise that PKS15 could be directly or indirectly involved in cell wall formation. This impact is significant, as the spore wall acts as a rigid frame that predetermines the size and shape of a fungal cell.

Generally, fungal walls are composed of chitin, β-1,3-glucan, β-1,6-glucan, glycoprotein, mannoprotein, and galactomannoprotein^17,18^, which could influence binding to host receptors and immune invasion^19,20^. For instance, *Candida albicans* mannoproteins^21^, *Paecilomyces farinosus* galactomannan^22,23^ and *Nomuraea rileyi* galactose induce opsonization of fungal cells by insect hosts. Changes in the wall surface may therefore affect host–pathogen interactions, as seen when the loss of chitin and β-1,3 glucan increases the ability of fungal cells to escape insect immune responses^24^. In mycobacteria, the type I polyketide synthase gene *pks13* is important for biosynthesis of mycolic acid, a major wall component of such pathogenic bacteria, and its deletion results in a change in the cell envelope structure^25^. Mycolic acid-containing glycolipids are also important for virulence in mice^26^. These observations further support the concept that *pks15* plays a role in cell wall formation and subsequently affects virulence in insect hosts.

There are a few cases where fungal polyketides have been associated with insect virulence, sporulation or cell wall formation. For insect virulence, the red pigment oosporein has been reported to be important for *B. bassiana* virulence against insects, with the authors noting the effects of oosporein on host immunity modulation^27^ and limiting bacterial growth after host death^28^. The Δ*pksA* mutant of the saprophytic fungus *Aspergillus parasiticus*, which is deficient in biosynthesis of dothistromin (a polyketide structurally related to aflatoxin), was also reported to have reduced sporulation^29^. Notably, sporulation rates were only a third that of the *A*. *parasiticus* wild type, similar to the sporulation impairment seen for *B. bassiana* Δ*pks15*. In two ascomycetes, *Sordaria macrospora* and *Neurospora crassa*, mutants lacking the putative dehydrogenase gene *fbm1*, which is in a polyketide biosynthesis cluster, were impaired in sexual development, particularly in perithecia formation^30^. In our current study, the role of PKS15 in cell wall integrity was clearly demonstrated by targeted gene disruption^4^ and genetic complementation. We therefore hypothesize that PKS15 and its metabolite might have a mechanistic role in cellular signaling during developmental processes, including sporulation, germination and spore wall formation, some of which clearly affect insect virulence. Alternatively, the PKS15 metabolite could directly be a spore wall component. Nonetheless, these two possibilities remain hypothetical and require further experimental proof. While involvement of non-reducing PKSs in cell wall formation have been reported^6–10,31^. Our study is the first to report a role for a reducing PKS in cell wall formation. Cell wall alteration seen for Δ*pks15* could account for the impaired phagocytic survival and consequently affect virulence against insects.

CRISPR/Cas9 has recently become one of the most popular molecular tools for genome editing due to its extraordinary capability for modifying genomes of various organisms. However, application of CRISPR/Cas9 editing to filamentous fungi such as *B. bassiana* is still in a preliminary stage. To our knowledge, there is only one prior study using CRISPR/Cas9 for genome editing in *B. bassiana*^32^. Here, we expand the great potential of CRISPR/Cas9 in fungal molecular genetic research by performing an in-*cis* genetic complementation of Δ*pks15* in *B. bassiana* using this technique. Interestingly, we successfully used a CRISPR/Cas9 vector previously developed for *A. Aculeatus*^13^ without any modification, despite the phylogenetic distance between *A. aculeatus* and *B. bassiana* and the rare occurrence of homologous recombination in filamentous fungi. This success may also be attributed to consideration of circumstances affecting CRISPR/Cas9-driven HDR, as DNA donor characteristics such as DNA form (circular versus linear) and arm sizes affect the repair success rate. For instance, as linear DNA donors are prone to degradation and are considered less efficient than circular DNA donors^33^, we used a circular DNA donor as the template for HDR. In contrast, attempts with linear donor DNA of similar length were unsuccessful in driving HDR in this fungus (unpublished data). We also used longer flanking regions to increase the efficiency of homologous recombination, as arms 1 kb or longer on either side of a target gene are generally used to facilitate successful recombination in filamentous fungi^34^. Our study demonstrated that a 600-bp arm was also sufficient to mediate HDR in *B. bassiana*. This finding is in agreement with a previous report that successfully used 250-bp arms for homologous recombination via CRISPR/Cas9^32^. Our study therefore supports the use of CRISPR/Cas9 as a powerful tool for site-specific mutagenesis and genetic complementation in this fungal entomopathogen.

## Methods

### Strains, culture conditions and genomic DNA preparation

*Beauveria bassiana* strain BCC 2660 was obtained from Thailand’s BIOTEC Culture Collection, and the *pks15* mutant was previously generated to contain a disruption in *pks15*, rendering the PKS gene nonfunctional^4^. The two strains were grown on half-strength potato dextrose agar (PDA; Difco, USA) at 25 °C for 5–7 days for the production of conidia. To produce blastospores, fungal conidia were inoculated in Sabouraud dextrose broth (Difco) supplemented with 1% yeast extract (SDY), and shaken at 150 rpm, 25 °C for 2 days. *Escherichia coli* strain DH5α was employed for plasmid propagation. For genomic DNA preparation, all fungal strains were grown in SDY as described above. All the cells were collected by centrifugation at 7,500 × *g* and fungal genomic DNA was extracted as previously described^7^.

*Acanthamoeba castellanii*, obtained from the Faculty of Tropical Medicine, Mahidol University, Thailand, was grown in peptone-yeast extract-glucose (PYG) broth as previously described^4^.

### General molecular methods

Standard molecular techniques were performed^35^ for plasmid purification, restriction enzyme analysis and DNA ligation. For PCR amplifications, we used the following thermal cycling program: 5 min at 95 °C; 35 cycles of 15 s at 95 °C, 25 s at 55 °C, and 1–5 min at 72 °C depending on the length of expected PCR products (1 min for a 1-kb product, 2 min for a 2-kb product and 5 min for products 5 kb or longer); and 5 min at 72 °C.

Ligated fragments were transformed into *Escherichia coli* DH5α, and bacterial clones were screened by PCR amplification with the primers CSN389 and CSN390 to verify the presence of the sgRNA insert. Clones with the correct insert was inoculated in LB and the plasmids were extracted using the GeneJet Plasmid Miniprep Kit (Thermo Fisher Scientific, USA). The recombinant plasmids were submitted for DNA sequencing (Macrogen, South Korea).

### Construction of bar-targeting CRISPR/Cas9 vector (pCas9sgBar)and DNA donor

The CRISPR/Cas9 vector containing sgRNA and Cas9 elements was constructed to generate a DNA DSB at the *bar* cassette of the Δ*pks15* mutant. Candidate protospacer sequences targeting *bar* gene 5′-CCACCTGCTGAAGTCCCTGGAGG-3′ (PAM site underlined) was generated using the sgRNA analysis tool available on the ge-CRISPR website (https://bioinfo.imtech.res.in/manojk/gecrispr/index.php). The CRISPR/Cas9 vectors pFC332 (harboring the hygromycin resistance gene) and pFC334, kindly gifted by Nødvig et al.^13^, were employed as the backbone vector and sgRNA cassette template, respectively, for the construction of pCas9sgBar. Two fragments for expression of *bar*-specific sgRNA cassette were amplified using pFC334 as the template. The first 545-bp fragment were amplified with primers CSN389 Fwd (5′-GGGTTTAAUGCGTAAGCTCCCTAATTGGC-3′) and Bar3 RV (5′-AGCTTACUCGTTTCGTCCTCACGGACTCATCAG CCACCTCGGTGATGTCTGCTCAAGCG-3′; the *bar* sequence is underlined). The second 424-bp fragment was amplified with primers Bar3 Fwd (5′-AGTAAGCUCGTCCCACCTGCTGAAGTCCCTGG GTTTTAGAGCTAGAAATAGCAAGTTAAA-3′; the *bar* sequence is underlined) and CSN390 RV (5′-GGTCTTAAUGAGCCAAGAGCGGATTCCTC-3′). PCR amplification was accomplished using recombinant Taq polymerase (Thermo Fisher Scientific). The mentioned two fragments of sgRNA were cloned into two distinct sites *Pac*I/*Nt*.*Bbv*CI of pFC332 using Uracil-Specific Excision Reagent (USER) fusion (New England Biolabs (NEB), USA).

To construct the circular DNA donor pCR-PKS15-1 k for genetic complementation of the Δ*pks15* mutant, a 1.0-kb *pks15* fragment was cloned into the TA vector pCR™ 2.1 (Thermo Fisher Scientific) using PCR amplification of *B. bassiana* BCC 2660 genomic DNA with the primers PKS15-start (5′-ATGCTCATCGACAAAATGGAG-3′) and PKS15-1182R (5′-CAGCATGAGAGTAGACTTGATGAC-3′). This circular donor DNA has sequences complementary to the 621-bp left arm and 561-bp right arm flanking the *bar* cassette in the Δ*pks15* mutant. PCR amplification was performed using Q5 High-Fidelity DNA polymerase (NEB).

### PEG-mediated protoplast transformation of ∆*pks15* mutant

We performed PEG-mediated protoplast transformation as previously described^8^. For complementation of Δ*pks15*, *pks15* mutant was transformed with pCas9sgBar and/or the donor DNA pCR-PKS15-1 k. For each transformation, a conidial suspension, as harvested from a 7-day old PDA culture, was used as inoculum of 50 ml PDB and shaken at 150 rpm, 25 °C for 16 h. Young mycelia were harvested by centrifugation at 3,000 × *g*. To generate protoplasts, the mycelia were incubated with the wall-lysing enzyme VinoTaste® Pro (Novozymes, Denmark) at a final concentration of 35 mg ml^−1^ in 1.2 M MgSO_4_, 10 mM sodium phosphate buffer, pH 5.8. The protoplasts were collected by centrifugation at 1,500 × *g*, 4 °C for 15 min for a pellet of approximately 10^8^ cells. The pellet was gently mixed by pipetting with 160 μl of STC (1.2 M sorbitol, 10 mM CaCl_2_, 10 mM Tris–HCl, pH 7.5) and 40 μl of transformation buffer (50% w/v polyethylene glycol (PEG)-4,000 (Sigma-Aldrich, USA) and 20 µg of pCas9sgBar (*bar*-specific sgRNA) and/or 15 µg of pCR-PKS15-1 k (donor DNA). Next, 50% w/v PEG was gently mixed to the protoplast-DNA solution for a total volume of 1,200 µl and left to incubate at room temperature for 20 min. The protoplast solution was then mixed with 8 ml of STC and incubated at room temperature for another 20 min. The mixture was subsequently centrifuged at 1,500 × *g*, 4 °C for 15 min to collect transformants. The pellet was gently resuspended in 1 ml of PDB supplemented with 1 M sucrose (QReC, New Zealand), and incubated at 28 °C with gentle shaking (70–80 rpm) for 30 min. Finally, 500 μl of the transformation mixture was added to 25 ml of molten PDA supplemented with 1 M sucrose and 200 mg l^−1^ hygromycin B and poured onto a 150 mm-diameter petri dish. The culture was incubated at 28 °C until fungal colonies appeared (approx. 7–14 days).

### Molecular analyses of Δ*pks15* isolates complemented with *pks15*

To determine the presence or loss of the *bar* cassette in the *pks15* locus of Δ*pks15* isolates complemented with *pks15*, transformed fungi were grown in SDY broth, and genomic DNA was extracted as described above.

We checked for the presence of the *bar* cassette in the *pks15* locus using PCR analysis. For the first two PCR amplifications, two primer pairs: Bar-100F (5′-AAGCACGGTCAACTTCCGTAC-3′) and PKS15-1182R (5′-CAGCATGAGAGTAGACTTGATGAC-3′); and PKS15-start (5′-ATGCTCATCGACAAAATGGAG-3′) and Bar-360R (5′-CTTCAGCAGGTGGGTGTAGA-3′) were used to amplify 1,105-bp and 1,375-bp fragments of the *pks15*-*bar-*boundary regions, respectively. For the third PCR amplification, the primer pair PKS15-minus-895F (5′-AATCTGCAGTGCCGAAGCTCCTAACCTCAG-3′) and PKS15-2200R (5′-GCTTATCAATGTGAGCCTCGTC-3′) were used to amplify a 3,095-bp *pks15* fragment from the wild type and complemented isolates and a 4,176-bp fragment from the Δ*pks15* mutant. PCR amplification was performed as described above. Lastly, the PCR fragments were submitted for DNA sequencing (Macrogen).

For Southern analysis, 25 mg of each genomic DNA from *B. bassiana* wild type, *Δpks15* mutant or complemented isolate G6.6 were digested to completion with *Eco*RI. Digested genomic DNAs were subjected to electrophoresis in a 1% agarose gel, and transferred and cross-linked to a nylon membrane (Hybond N+; GE Healthcare Bio-Sciences, USA) according to the manufacturer’s instructions. To prepare a DNA probe for Southern analysis, a 1.4-kb-long *pks15* fragment was amplified with the primers PKS15-minus 925F (5′-TCAAGCTTGCCCGTCACTTG-3′) and PKS15-480R (5′-CAAGCTTCCGGTACGATAGTC-3′), and 100 ng of this DNA fragment was non-radioactively labelled using North2South™ Biotin Random Prime Labeling Kit (Thermo Fisher Scientific). Membrane hybridization and signal detection was performed using North2South™ Chemiluminescent Hybridization and Detection Kit (Thermo Fisher Scientific) according to the manufacturer’s instructions. The nylon membrane (Amersham Hybond-N + GE Healthcare, Life Sciences) was hybridized with the biotinylated-*pks15* probe at 55 °C overnight. After a high stringency wash with 2× saline sodium citrate (SSC)/0.1% sodium dodecyl sulfate (SDS) at 55 °C, the membrane was incubated with a streptavidin–horseradish peroxidase conjugate solution. The hybridized membrane was analysed by a CCD high-resolution chemiluminescence detection system (ChemiDoc™ XRS + System, Bio-Rad, USA).

### Insect bioassay and determination of sporulation and germination

To assess insect virulence, conidia of the *B. bassiana* wild type, Δ*pks15* mutant, and a complemented isolate were separately harvested in saline (0.85% NaCl), and the density was adjusted to 1 × 10^5^ cells ml^−1^ using a haemocytometer. Third- to fourth-instar beet armyworm (*Spodoptera exigua*) larvae were injected with a 3-µl conidial suspension of one of the three fungal strains using a specialized 33-gauge needle-syringe set (Hamilton, USA). Injected larvae were transferred individually into a 24-well plate and fed with an artificial medium^6^. Saline-injected worms were used as controls. Ten insect larvae were treated for each fungal strain and the saline control. The experiment was repeated three times. Larval mortality was determined on days 1–7 after fungal inoculation. Mean lethal time (LT_50_) was determined using Probit analysis (SPSS package version 11.5).

To assess sporulation, production of conidia and blastospores were determined on 5-day-old half-strength PDA and in 2-day-old SDY cultures, respectively. PDA and SDY cultures were prepared as described above using 100 µl and 1 ml of conidial suspension at 1 × 10^7^ conidia in sterile water, respectively. To determine conidial germination, conidia were harvested from PDA as described above and resuspended in 5% (v/v) PDB in sterile water. Germination percentage was determined after incubation for 20 h. There were three replicates for each strain, and the experiment was repeated twice.

### Phagocytic survival assay

Blastospores were assessed for phagocytic survival in *A*. *castellanii* as previously described^4^, with the yeast *S. cerevisiae* as a control. Briefly, the *B. bassiana* wild type and mutants were grown in SDY broth. *S. cerevisiae* and *A. castellanii* were grown in YPD broth and PYG broth respectively. After incubation for two days, all cells were collected by centrifugation, filtered (for removal of mycelia in fungal culture) and adjusted to 1 × 10^5^ cells ml^−1^. Each fungal strain was co-cultured with *A. castellanii* in 96-well plates at a 1:1 ratio for 72 h in order to determine post-challenge amoeboid survival rates and fungal CFUs. Determination of amoeboid survival rates was performed as previously described^4^. Trypan blue dye was used to determine the viability of amoebae. The experiment was repeated twice.

### Ultrastructural characterization of cell wall surface

For scanning electron microscopy (SEM), PDA-grown conidia were fixed with 2.5% glutaraldehyde (Electron Microscopy Sciences (EMS), USA) and 2% paraformaldehyde (EMS) in phosphate buffer (0.1 M KH2PO4 and Na2HPO4, pH 7.2), followed by fixation in 1% OsO4 (EMS), as previously described^36^. The cells were then dehydrated with an ethanol gradient from 30, 50, 70, 80, 90, 95, to 100% ethanol, then critical-point dried using a CO_2_ drier model HCP-2 (Hitachi, Japan) and sputter-coated with gold-platinum with the Q150R Au/Pt coater (Quorum Technologies, UK). Photographs were taken with SEM model FE-SEM SU-5000 (Hitachi).

For atomic force microscopy (AFM), conidia were prepared by growing fungal strains on PDA for 7 days before conidia were dislodged with 10 ml of sterile water. Conidial suspensions were filtered through eight layers of cheesecloth and the flow-through centrifuged at 13,700 × g for 2 min, rinsed with PBS buffer twice, and finally resuspended in 1 ml of PBS buffer. Sixty microliters of the conidial suspension were dropped on a membrane filter with 1.2 µm pore size (MF-Millipore, Germany) and air-dried overnight. Each conidial sample was mounted on a glass slide and subjected to atomic force microscopy (AFM).

AFM imaging was performed with the NanoWizard 3 AFM system (JPK Instruments, Bruker, USA) mounted on an isolation platform to minimize surrounding noise. Amplitude and phase images were collected in the AC (non-contact) mode under ambient conditions. An amplitude image records the amplitude changes of the oscillating cantilever during the scan, thus reflecting the vertical distance (Z-direction) of the sample. A phase image records the phase difference between the cantilever input signal cycle and the responsive signal cycle, reflecting the mechanical properties of the sample such as stiffness, and adhesion. The AFM cantilever for this study was a non-contact mode silicon AFM cantilever (product no. ACTA, AppNano, USA) with a nominal spring constant of 37 N m^−1^ and a tip radius of curvature of approximately 6 nm. The images were recorded at a scanning speed of 0.5 Hz at different resolutions, including 256 × 256 pixels, or 512 × 512 pixels.

### Cell wall staining

*Beauveria bassiana* conidia and blastospores were collected from 7-day-old PDA and 2-day-old SDY cultures, respectively, and rinsed once with PBS. Spores were stained with concanavalin A conjugated with the fluorophore FITC (Sigma-Aldrich) and calcofluor white (Sigma-Aldrich). For conA staining, spores were fixed with 3.7% formaldehyde (in 100 µl) for 20 min and rinsed with PBS once. Then, the spores were incubated in 20 µl of 1 mg ml^−1^ conA in PBS (pH 7.4) for 60 min and rinsed once with PBS. For calcofluor staining, spores were incubated in a 1:1 mixture of 5 mg ml^−1^ calcofluor white in PBS (pH 7.4) and 1 M KOH in a total volume of 200 µl. The cell staining mixture was incubated at room temperature for 2 min. Stained spores were collected by centrifugation at 6,729 × *g* for 1 min and rinsed once with PBS.

### Confocal and fluorescence microscopy and cell measurements of stained fungal spores

The concanavalin A- and calcofluor-stained fungal spores were wet-mounted onto glass slides and covered with No.1 (0.13-0.16 um thickness) cover glass (BRAND, Germany). Fluorescence images were acquired by the Nikon Eclipse Ti-2E using the Nikon Plan Apo VC 100 × oil objective lens (numerical aperture (N.A.) = 1.40). Images were captured at 2,880 × 2,048 pixels in TIFF format.

For confocal laser scanning microscopy, we used the Olympus FV1000 confocal laser scanning system configured with the Olympus IX81 inverted microscope with the Olympus UIS2 UPLSAPO 100 × oil objective lens (N.A. = 1.4). All images were recorded at a resolution of 1,600 × 1,600 pixels using a 100 × oil immersion objective lens with a 3 × digital zoom. Parameters for calcofluor staining images were as follows: laser power, 20%; HV, 400–500; gain, 1 and pinhole, 150–155 (auto). Parameters for concanavalin A staining images were as follows: laser power, 30%; HV, 600–650; gain, 1–3 and pinhole, 150–155 (auto). For each image, the offset voltage was adjusted until the background appeared black or nearly so. Kalman filtering (n = 6) was generally used to improve the signal-to-noise ratio of images. All images were captured as TIFF files.

Spore sizes and shapes were compared using two dimensional image analysis of conA- or calcofluor-stained spores taken by the Eclipse Ti-2E and the NIS-Elements software (Nikon). For determination of spore sizes, the ‘5 Point Ellipse’ feature for area measurement of NIS-Elements was manually used to mark the boundary of a conidium and the ‘Auto Detect’ feature was used to mark the boundary of a blastospore (Supplemental Fig. S3). Consequently, ‘size’ was automatically determined as ‘area’ by the software. Budding blastospores were excluded from the analysis.

In NIS-Elements, shape factor, calculated using the formula shape factor = 4πA/P^2^ where A = area and P = perimeter, was used to determine whether a spore is circular or elliptical in shape. The shape factor value of a circle equals 1.00, whereas values less than 1.00 indicates an ellipse.

### Statistical analyses

All experiments in this study were repeated two or three times. There were three replicates for each fungal strain in a given experiment. Data were analyzed for statistical significance using ANOVA in SPSS package version 11.5 and the student’s *t*-test. LT_50_ was determined using Probit analysis in the SPSS package.

## Supplementary information

Supplementary Figures.
