# Supplementary material for: The polyketide synthase PKS15 has a crucial role in cell wall formation in Beauveria bassiana
Source: Sci Rep. 2020 Jul 28;10:12630. doi: 10.1038/s41598-020-69417-w (PMC7387532; doi:10.1038/s41598-020-69417-w)
Supplement: Supplementary file 1 — Supplementary Figures. [file 41598_2020_69417_MOESM1_ESM.pdf]

Supplementary Information

The polyketide synthase PKS15 has a crucial role in cell wall formation in *Beauveria bassiana*

Somsiri Udompaisarn[1], Wachiraporn Toopaang[1], Udom Sae-Ueng[1], Chettida Srisuksam[1], Nuchnudda Wichienchote[1], Rudsamee Wasuwan[1], Nur Amalina Shamsun Nahar[3], Morakot Tanticharoen[2] and Alongkorn Amnuaykanjanasin[1,*]

[1] National Center for Genetic Engineering and Biotechnology (BIOTEC), National Science and Technology Development Agency (NSTDA), 113 Thailand Science Park, Paholyothin Rd., Tambon Khlong Nueng, Amphoe Khlong Luang, Pathum Thani 12120, Thailand

[2] School of Bioresources and Technology, King Mongkut's University of Technology Thonburi, Bangkok 10140, Thailand

[3] Temasek Polytechnic, 21 Tampines Avenue 1, Singapore 529757, Singapore

[*] Correspondence: alongkorn@biotec.or.th

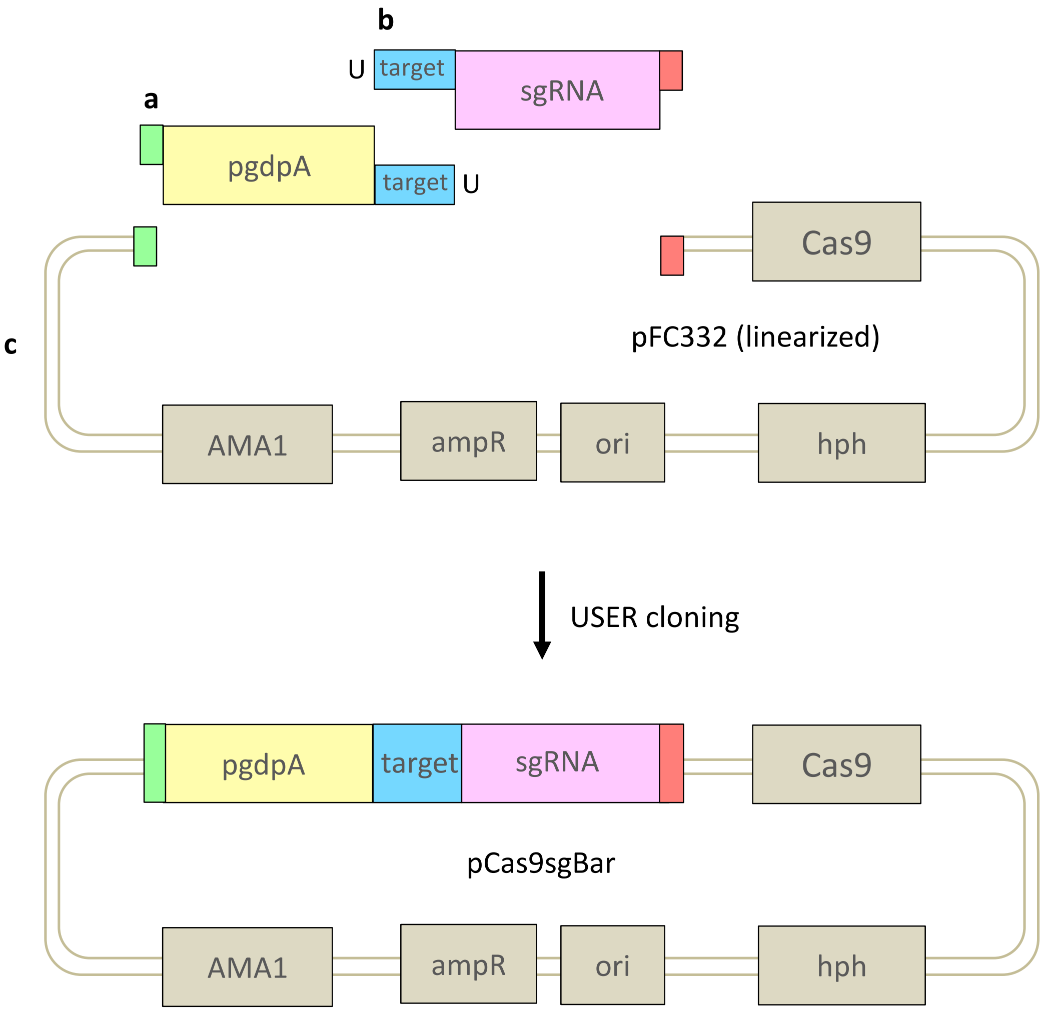

Supplemental Figure S1. Schematic representation of the pCas9sgBar plasmid construction. Detailed cloning protocols are described previously (Nodvig et al., 2015). Briefly, the guide RNA is assembled by a fusion of two fragments in order to introduce "our gene-specific guide RNA (sgRNA) sequence" in the vector. The gene-specific guide RNA sequence is incorporated in the primers used for the vector construction. The vector pFC334 was used as the template to amplify (a) pgdpA tRNA promoter element and (b) sgRNA expression cassette. Each PCR fragment was amplified by primers containing 25 nucleotides overhang (=target) and followed by a uracil base (U). Uracil DNA glycosylase and DNA glycosylase-lyase Endonuclease VIII (USER Enzyme) was used for elimination of the uracil bases in the PCR fragments. This enzyme digestion generated complementary overhangs at the ends of all three fragments, which facilitated the fusion of the two PCR fragments and the linearlized vector pFC332 by USER cloning in one step. (c) The vector backbone for construction of pCas9sgBar is derived from the pFC332. Sticky ends for USER cloning are achieved by restriction digestion of PacI (green) and Nt.BbvCI (red) sites in pFC332. It is noted that AMA1 is a plasmid replicator, originally reported in *Aspergillus nidulans* (Aleksenko and Clutterbuck, 1997) and recently used in Nodvig et al. (2015). In principle, an AMA1-containing plasmid could be easily lost when there is no selection. However, the autonomous replication of such plasmid remain to be proven in each fungus tested.

Supplemental Figure S2. Sequence alignment of the *pks15* locus of *B. bassiana* wild type (Query) and that of the complemented isolate G6 (Sbjct)

```
Query  1     ATGCTCATCGACAAAATGGAGACGCCCACACCTCTTGCCATTGTCGGCATGGCCTGCCGC  60
             ||||||||||||||||||||||||||||||||||||||||||||||||||||||||||||
Sbjct  1     ATGCTCATCGACAAAATGGAGACGCCCACACCTCTTGCCATTGTCGGCATGGCCTGCCGC  60

Query  61    ACGTCCGGTGATGTGAGGACAATCGATGAGTTTTGGACTATGCTGTCTCGGTCCCGACAC  120
             ||||||||||||||||||||||||||||||||||||||||||||||||||||||||||||
Sbjct  61    ACGTCCGGTGATGTGAGGACAATCGATGAGTTTTGGACTATGCTGTCTCGGTCCCGACAC  120

Query  121   GGTTCCGGCCCTATTCCCAAGAGTCGATACAATGCCGAGGCTTACTACCACCCGAATCCG  180
             ||||||||||||||||||||||||||||||||||||||||||||||||||||||||||||
Sbjct  121   GGTTCCGGCCCTATTCCCAAGAGTCGATACAATGCCGAGGCTTACTACCACCCGAATCCG  180

Query  181   CAAAAGAGAGGCACCTTCAACCAAGTTGGTGGCTACTTCATCGATAGAGATTTCTCCGTC  240
             ||||||||||||||||||||||||||||||||||||||||||||||||||||||||||||
Sbjct  181   CAAAAGAGAGGCACCTTCAACCAAGTTGGTGGCTACTTCATCGATAGAGATTTCTCCGTC  240

Query  241   TTTGACGCGCCTTTCTTCAACATTACCAAACAAGAAGCCAGTTCAATGGGTAAGTACTGC  300
             ||||||||||||||||||||||||||||||||||||||||||||||||||||||||||||
Sbjct  241   TTTGACGCGCCTTTCTTCAACATTACCAAACAAGAAGCCAGTTCAATGGGTAAGTACTGC  300

Query  301   CAGATATTCATATGGCCACTTCGCCGGTGCTAATGCGCCTCGTCAGACATCAACCAGAGA  360
             ||||||||||||||||||||||||||||||||||||||||||||||||||||||||||||
Sbjct  301   CAGATATTCATATGGCCACTTCGCCGGTGCTAATGCGCCTCGTCAGACATCAACCAGAGA  360

Query  361   CAACTTCTCGAATGCACATACGAAGCCCTGGAAAATGCTGGTCTACCCAAGGGTAAGATC  420
             ||||||||||||||||||||||||||||||||||||||||||||||||||||||||||||
Sbjct  361   CAACTTCTCGAATGCACATACGAAGCCCTGGAAAATGCTGGTCTACCCAAGGGTAAGATC  420

Query  421   TCCGGCGAAAAGATGGGTGTCTTCATTGGAACGAAACGAGCCGACTATCGTACCGGAAGC  480
             ||||||||||||||||||||||||||||||||||||||||||||||||||||||||||||
Sbjct  421   TCCGGCGAAAAGATGGGTGTCTTCATTGGAACGAAACGAGCCGACTATCGTACCGGAAGC  480

Query  481   TTGCAGGACCTCAACCAAGTCGAAATGTTCGAAGCTACCAGTGGCCACAGCTCCATTCAG  540
             ||||||||||||||||||||||||||||||||||||||||||||||||||||||||||||
Sbjct  481   TTGCAGGACCTCAACCAAGTCGAAATGTTCGAAGCTACCAGTGGCCACAGCTCCATTCAG  540
```

```
Query  541   GCTGGTCGTATCTCATACTATTTCAACTTGAATGGCCCATCCTTTTCGGTGGACACGGCG  600
             ||||||||||||||||||||||||||||||||||||||||||||||||||||||||||||
Sbjct  541   GCTGGTCGTATCTCATACTATTTCAACTTGAATGGCCCATCCTTTTCGGTGGACACGGCG  600

Query  601   TGTTCCTCTAGTTTGCACGCTATGCATGCTGCTGTGCAGAGCATCCGAAGTGGCGACTGC  660
             ||||||||||||||||||||||||||||||||||||||||||||||||||||||||||||
Sbjct  601   TGTTCCTCTAGTTTGCACGCTATGCATGCTGCTGTGCAGAGCATCCGAAGTGGCGACTGC  660

Query  661   GACTCTGCCATTGTTGCTGCCAGCAATCTATACATGAATCCGGGCGATCTAATCCAAATG  720
             ||||||||||||||||||||||||||||||||||||||||||||||||||||||||||||
Sbjct  661   GACTCTGCCATTGTTGCTGCCAGCAATCTATACATGAATCCGGGCGATCTAATCCAAATG  720

Query  721   TCTATGCTGGGTCTCTTTACCCCAACAGGTAAGACTTACGCCTTCGATCACAGAGGCCAA  780
             ||||||||||||||||||||||||||||||||||||||||||||||||||||||||||||
Sbjct  721   TCTATGCTGGGTCTCTTTACCCCAACAGGTAAGACTTACGCCTTCGATCACAGAGGCCAA  780

Query  781   TCAGGCTTTGCATGCGGTGAAGGCGTCGGCTGTGTTGTTATCAAGCCTCTCGACCAGGCC  840
             ||||||||||||||||||||||||||||||||||||||||||||||||||||||||||||
Sbjct  781   TCAGGCTTTGCATGCGGTGAAGGCGTCGGCTGTGTTGTTATCAAGCCTCTCGACCAGGCC  840

Query  841   CTGCGTGATAATGACAAGATTCGCTCCGTCATTGTCAACACGGGAATCAACCAGGACGGC  900
             ||||||||||||||||||||||||||||||||||||||||||||||||||||||||||||
Sbjct  841   CTGCGTGATAATGACAAGATTCGCTCCGTCATTGTCAACACGGGAATCAACCAGGACGGC  900

Query  901   AAAACCGTCGGCTTGACTTCACCGAGTGCCAGACAGCAGGAGAACCTCATGCGCGAGGTC  960
             ||||||||||||||||||||||||||||||||||||||||||||||||||||||||||||
Sbjct  901   AAAACCGTCGGCTTGACTTCACCGAGTGCCAGACAGCAGGAGAACCTCATGCGCGAGGTC  960

Query  961   TATGCTCGCGCCCACATTAGCCCAAGCGACGTCGGCTTTGTCGAAGCCCACGGCACGGGT  1020
             ||||||||||||||||||||||||||||||||||||||||||||||||||||||||||||
Sbjct  961   TATGCTCGCGCCCACATTAGCCCAAGCGACGTCGGCTTTGTCGAAGCCCACGGCACGGGT  1020

Query  1021  ACAAAGGTTGGCGATCCTTTGGAGGCCACCGCAATTCACAACTTCTTTGGTCAAGGCCGC  1080
             ||||||||||||||||||||||||||||||||||||||||||||||||||||||||||||
Sbjct  1021  ACAAAGGTTGGCGATCCTTTGGAGGCCACCGCAATTCACAACTTCTTTGGTCAAGGCCGC  1080


Query  1081  ACTAAGCGTTCGCCTCTATACCTTGGCTCAGTGAAGACCAACATCGGCCATCTGGAGAAT  1140
             ||||||||||||||||||||||||||||||||||||||||||||||||||||||||||||
```

```
Sbjct  1081  ACTAAGCGTTCGCCTCTATACCTTGGCTCAGTGAAGACCAACATCGGCCATCTGGAGAAT  1140

Query  1141  GCCAGTGGTTTGCTCTCCGTCATCAAGTCTACTCTCATGC  1180
             ||||||||||||||||||||||||||||||||||||||||
Sbjct  1141  GCCAGTGGTTTGCTCTCCGTCATCAAGTCTACTCTCATGC  1180
```

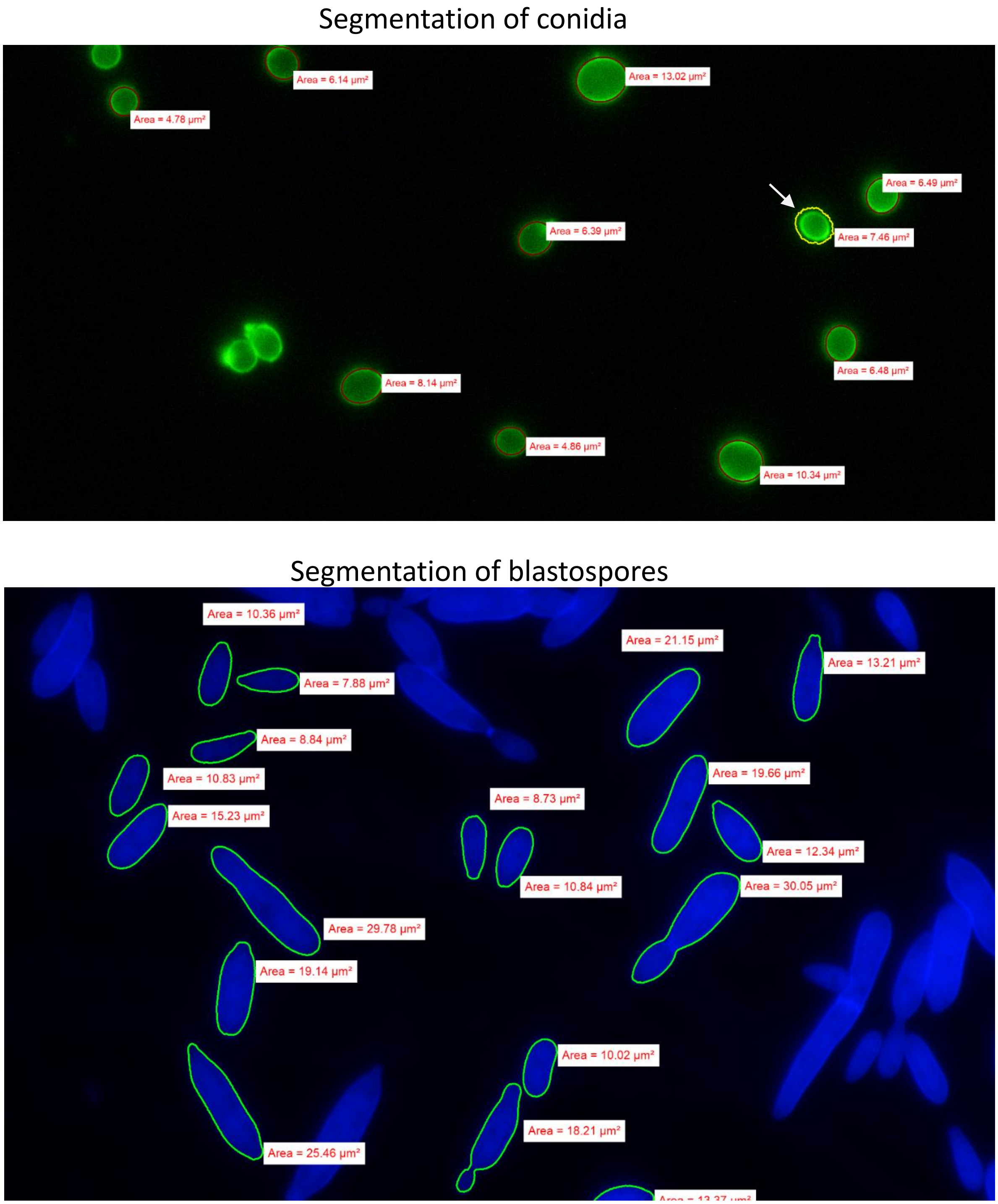

Supplemental Figure S3. Determination of spore size and shape in 2D images. A. Segmentation of conidia, stained by FITC-tagged concanavalin A. The '5 Point Ellipse' feature in the area measurement of the NIS-Elements was manually used to mark the boundary of a conidium. **B.** Segmentation of blastospores, stained by calcofluor white. The 'Auto Detect' feature was used to indicate the boundary of a blastospore. Then, 'size' was automatically determined as 'area' by the software. The budding blastospores were excluded in the analysis. It is noted that if the 'Auto Detect' feature was used to mark the boundary of a conidium (indicated by an arrow), the segmentation was not accurate due to the fluorescence appearing outside the conidium.
